# Childhood esophageal achalasia: Case report from Afghanistan with literature review

**DOI:** 10.1016/j.ijscr.2022.107112

**Published:** 2022-05-02

**Authors:** Turyalai Hakimi, Ramazan Karimi

**Affiliations:** Department of Pediatric Surgery, Kabul University of medical science, Maiwand teaching hospital, Kabul, Afghanistan

**Keywords:** Achalasia, Motility, Dysphagia, Regurgitation, Esophagomyotomy, Fundoplication, GI, Gastrointestinal, LES, Lower esophageal sphincter, GERD, Gastrointestinal reflux disease, END, Endoscopic pneumatic dilatation, HRM, High-resolution manometry, HM, Heller myotomy, POEM, Peroral endoscopic myotomy

## Abstract

**Introduction and importance:**

Esophageal achalasia is a motility disorder of the esophagus with unknown etiology characterized by the failure of lower esophageal sphincter relaxation. Diagnosis is made by barium esophagography, endoscopy, and esophageal manometery. Heller Esophagomyotomy along with Dor's fundoplication is the treatment of choice. Persisting undiagnosed cases may lead to malnutrition.

**Case presentation:**

We present a case of an 8-year-old child suffering from dysphagia and regurgitation. The child was misdiagnosed and maltreated for the suspicion of respiratory tract and gastrointestinal problems in the local clinics. During this time, he remained unresponsive to the mentioned treatments, and the local physician advised him to have an upper gastrointestinal (GI) endoscopy, which revealed esophageal achalasia (EA).

On admission to our pediatric surgery ward, the patient had coexistent parotitis, which was treated conservatively. Following recovery, the patient was prepared for surgery and underwent esophageal myotomy along with Dor's fundoplication.

**Clinical discussion:**

Esophageal achalasia is rare in children, but poses major health challenges to children if left untreated. Symptomatic treatment may mask the actual picture of the problem and last for years. Following surgery and discharge from the hospital in a three-month follow-up interval of time, our patient exhibited full recovery, with gaining 4 kg weight.

**Conclusions:**

Respiratory and gastrointestinal conditions with similar signs and symptoms should always be considered in differential diagnosis of esophageal achalasia, especially where there is no direct access to a pediatric specialized complex. On-time evaluation and treatment will further prevent children from malnutrition in long-lasting undiagnosed patients.

## Highlights

• Esophageal achalasia is a motility disorder with unknown etiology.
• Esophageal achalasia is rare in children.
• Dysphagia and respiratory infection will mask the actual picture of the problem.
• Unprofessional symptomatic treatment for a long time is the main cause of undiagnosed patient and may lead to malnutrition.

## 1 Introduction

Achalasia cardia is defined as a neurodegenerative problem of the esophagus with unknown etiology, characterized by the failure of lower esophageal sphincter relaxation. It is uncommon in children under the age of 5, and the estimated annual incidence is 0.11/100000 live births [1], [2]. The problem is more prevalent in males and conditions like trisomy 21, congenital hyper ventilation syndrome, familial dysautonomia, Chagas' disease, eosinophilic esophagitis, glucocorticoid insufficiency, along with achalasia, alacrimia, and ACTH insensitivity syndrome (AAA) could also coexist [3].

Pathophysiologically, degeneration of the inhibitory myenteric plexus, which innervates the lower esophageal sphincter (LES), is implicated [4]. Abnormality in parasympathetic innervation may exist, but the etiology still remains unclear [5]. Common symptoms are dysphagia, vomiting, weight loss, and, atypically, the patient may suffer from pneumonia, nocturnal cough, aspiration, hoarseness, and feeding problems [3].

Achalasia is mostly misdiagnosed as gastrointestinal reflux disease (GERD). Most of the cases present as failure to thrive, eating problems, esophagitis, and asthma. Therefore, diagnosis is delayed until the child reaches 6–10 years of age, and for this reason, the patient is largely treated with prokinetics and antacids prior to diagnosis [2], [3]. This work has been reported in line with the SCARE 2020 criteria [6].

## 2 Case presentation

An 8-year-old child was referred by a local clinic from northern Afghanistan to our pediatric surgery department, suffering from dysphagia and regurgitation for 2 years. The child was born to a non-consanguineous couple, with a height of 135 cm and a weight of 21 kg. The ante-natal history was uneventful and the family history of the mentioned problem was negative for the patient's first and second-degree relatives.

The patient was treated symptomatically for the suspicion of having GI and respiratory tract infections with several visits by the local public and private clinics. Due to the deterioration of his problem and frequent visits by one of the physicians, he was advised to have an endoscopic analysis of the upper GI tract and was found to have achalasia. For this reason, the patient was referred to our hospital for definite treatment.

**Fig. 1:**
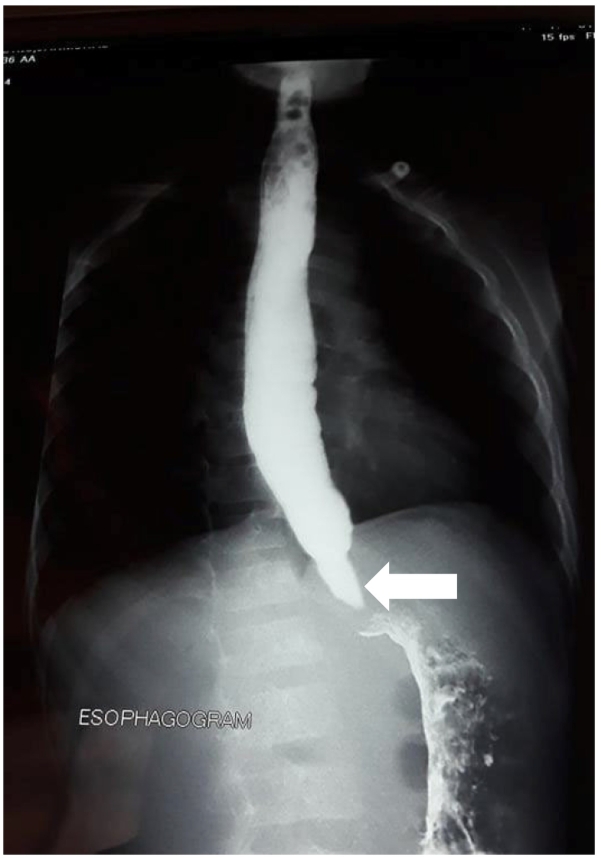
Barium esophagogram shows lower esophageal sphincter (LES) narrowing (Arrow, Bird beak sign).

The patient was admitted to our pediatric surgery ward. Physical exam revealed unilateral parotid gland inflammation (parotitis), loss of appetite but no hearing problems or testicular inflammation (orchitis). Lab tests showed white blood cell (WBC) count elevation and GI contrast study revealed LES narrowing (bird beak sign, Fig. 1) with its upper part compensatory dilatation, confirming the diagnosis of achalasia. Based on the patient's clinical picture, we postponed the operation and kept the patient under observation by initiating treatment for 10 days under the diagnosis of mumps.

**Fig. 2:**
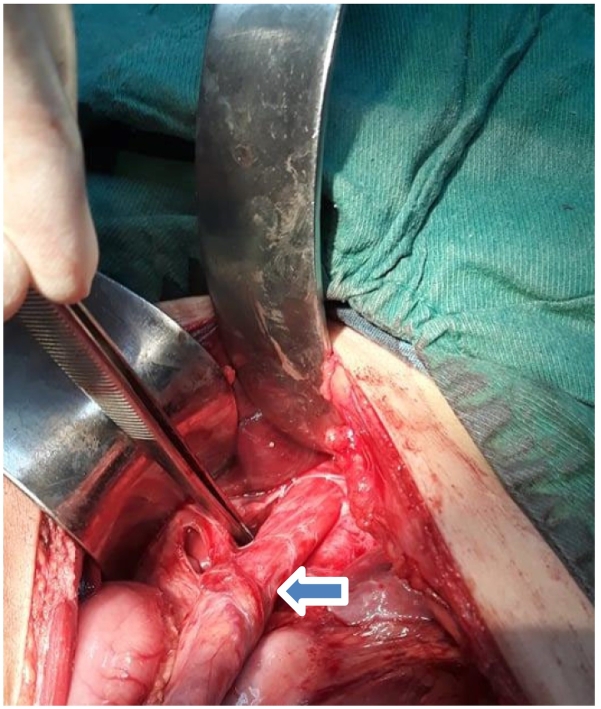
Mobilization of lower esophageal sphincter (LES).

**Fig. 3:**
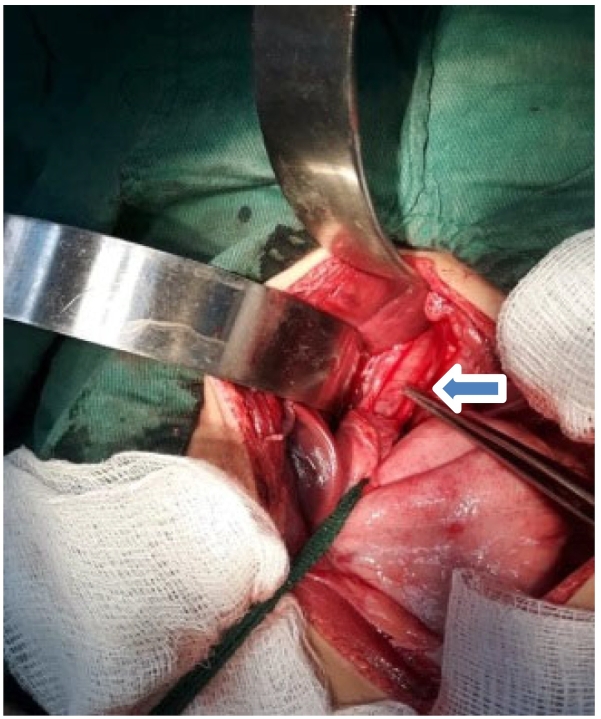
Complete mobilization of esophageal mucosa (Esophagomyotomy).

**Fig. 4:**
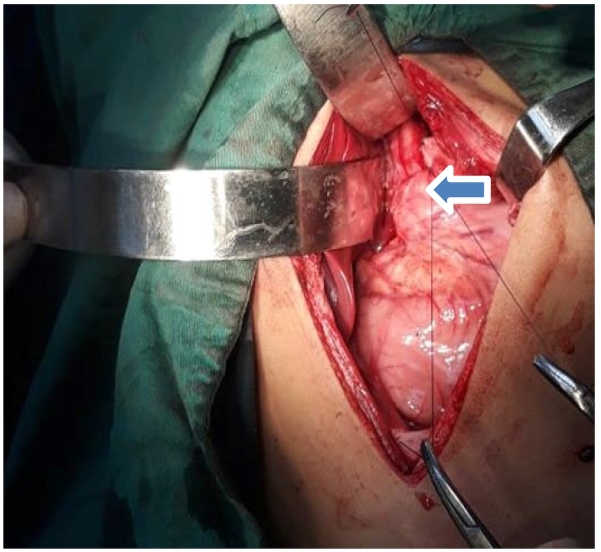
Dor's fundoplication procedure.

Following recovery from mumps, the patient was prepared for surgery. Our team approached the patient through the upper midline incision, mobilizing the LES (Fig. 2) and further up to the most dilated point. Esophagomyotomy (Fig. 3) along with Dor's Fundoplication was done (Fig. 4). On the 4th post-operative day, the patient was put on a liquid diet and gradually liquid to soft and then a regular food regimen. The patient's tolerance to food was appreciable, and on the 7th post-operative day, we discharged the patient in satisfactory condition. Following post-operative evaluation (every month for three consecutive months), the patient was symptom free and tolerated food with gaining 4 kg weight (a total net weight of 25 kg) during post-operative follow-up. Given the patient's symptom-free condition and food tolerability with no gastrointestinal problems, we omitted further investigation for re-evaluation and advised the patient to continue his normal life.

## 3 Discussion and conclusion

Esophageal achalasia is a rare motor disorder of the esophagus, resulting from degeneration or lack of ganglion cells in the lower esophageal muscle layer. The condition was first described by physician and neuroanatomist Sir Thomas Willis of England in 1674 [7], [8]. Esophageal achalasia is characterized by ineffective esophageal peristalsis due to increased resting tension and impaired swallow-induced relaxation of the lower esophageal sphincter [9], [10]. Rarely, a central nervous system lesion can cause LES achalasia. In some cases, infectious, environmental, and autoimmune factors are considered the main causes of these pathophysiologic changes, but most of the time it is idiopathic [11]. A number of studies failed to identify familial clustering of achalasia, but most of the achalasia cases may be as a result of consanguinity and may occur in siblings, suggesting autosomal recessive inheritance. Some authors argue about the genetic predisposition to achalasia [12], [13].

According to the literature, signs and symptoms occur to varying degrees ranging in decreasing order (dysphagia 90%, vomiting 76–91%, chest pain 17–95%, heartburn 27–42%) respectively [3], [14], [15]. Sometimes, achalasia may be complicated by esophagitis due to retained esophageal food or may be accompanied by malnutrition or respiratory symptoms [16]. Infants and toddlers present with choking, coughing, recurrent chest infections, feeding aversion, and failure to thrive. If left untreated, achalasia may lead to malnutrition and cachexia [17]. Average time from initial symptoms to diagnosis is 2 years but may be prolonged to 5 years, and due to oligosymptomatic progression in the initial phase and low prevalence of the disease, may last to 8.8 years [14], [15], [16].

Mostly, the diagnosis of achalasia is made by barium X-ray which is considered the first diagnostic approach with up to 95% effectiveness [18], [19]. It also helps patient monitor after the achalasia treatment [9], [20]. Gastroscopy may show residual food, mucosal changes due to chronic irritation and finding of tight LES which doesn't open by air insufflation [9]. Manometry remains the gold standard for the diagnosis of achalasia with up to 90% efficacy [19], [20]. It allows us to differentiate achalasia types according to Chicago classification [14], [21]. The positive predictive value of a barium swallow in comparison to esophageal manometry is as high as 96% [22]. Recent diagnostic tools such as high-resolution manometry (HRM) and multichannel intraluminal impedance pH monitoring (MII-pH) will provide further physiologic details in diagnostic doubts. It also gives details about achalasia subtypes (I-III) [23].

Different pharmacologic, endoscopic, and surgical treatment options are available. Intersphincteric Injection of botulinum is ineffective with high failure rate and requires repeated attempts in children. On the other hand, botulinum injection-induced scar formations around the cardia will make the myotomy procedure very difficult [24], [25]. Botulinum toxin injection is safe and somewhat simple, but the action is temporary and its usage in children is indefinite [9], [23], [24], [26], [27]. Efficacy of endoscopic pneumatic dilatation (END) is limited in children as 30–75% of children need subsequent surgery due to relapse of symptoms [21], [28], [29], [30].

Due to its efficacy and safety, Heller myotomy (HM) was a gold standard treatment [31]. Recently, peroral endoscopic myotomy (POEM) has been introduced as a minimally invasive treatment modality with similar efficacy to that of HM, but fewer complications [32].

The concomitant performance of fundoplication with HM is now controversial. Most authors consider fundoplication to be an essential part of the procedure [33], [34], [35]. The selection of the fundoplication technique is a personal conviction. Some authors suggest Toupet's or Dor's type, but some of them recommend 360° warp fundoplication [23], [36], [37], [38].

Our patient was misdiagnosed in early childhood and treated under the diagnosis of respiratory and gastrointestinal disease. Main symptoms were dysphagia and regurgitation. Our treatment option involved esophageal myotomy along with Dor's fundoplication. We allowed our patient to have his sips of water on the 4th post-operative day and on the next day liquid diet. On the seventh post-operative day he had his normal diet, so we discharged him with proper advice and followed him for three months fixed intervals evaluation (every month). Now he is symptoms free and tolerates all types of food normally. GI and respiratory conditions are two important entities which should always be considered in differential diagnosis. Long lasting undiagnosed pediatric achalasia cases may lead to malnutrition, so timely treatment is the integral part of management.

## Guarantor

The corresponding author is the guarantor of the work, having the responsibility of data access and controlling the decision to publish.

## Ethical approval and consent to participate

Not applicable.

## Consent to publish

Written informed consent was obtained from the patient for publication of this case report and accompanying images. A copy of the written consent is available for review by the Editor-in-Chief of this journal on request.

## Availability of data and materials

The datasets used in the current article, are available from the corresponding author on reasonable request.

## Funding

None.

## Provenance and peer review

Not commissioned externally peer-reviewed.

## CRediT authorship contribution statement

Turyalai Hakimi (TH) conceptualized the manuscript. TH and Ramazan Karimi (RK) designed the study. TH wrote the original draft. TH and RK performed the procedure data collection and analysis. TH wrote and edited the manuscript as well as supervised the entire study process. Both authors read and approved the final manuscript.

## Declaration of competing interest

The authors declare that they have no known competing financial interests of personal relationships that could have appeared to influence the work reported in this paper.
